# Natural bio-convective flow of Maxwell nanofluid over an exponentially stretching surface with slip effect and convective boundary condition

**DOI:** 10.1038/s41598-022-04948-y

**Published:** 2022-02-09

**Authors:** Fuzhang Wang, Shafiq Ahmad, Qasem Al Mdallal, Maha Alammari, Muhammad Naveed Khan, Aysha Rehman

**Affiliations:** 1 Nanchang Institute of Technology, Nanchang, 330044 China; 2 Department of Mathematics, Quaid-I-Azam University, Islamabad, Pakistan; 3 Department of Mathematical Sciences, UAE University, Alain, 15551 UAE; 4 Department of Mathematics, College of Science, King Saud University, P.O. Box 800, Al-Riyadh, 11421 Saudi Arabia; 5 Department of Mathematics, University of Gujrat, Gujrat, 50700 Pakistan

**Keywords:** Materials science, Mathematics and computing

## Abstract

The under-consideration article mainly focuses an unsteady three-dimensional Maxwell bio-convective nanomaterial liquid flow towards an exponentially expanding surface with the influence of chemical reaction slip condition. The feature of heat transport is achieving in the existenceof convective boundary condition and variable thermal conductivity. With the help of similarity variables, the flow form of equations is turned into a nonlinear form of coupled ODEs. The numerical solutions are calculated by adopting bvp4c function of MATLAB. Impact of distinct characteristics on the temperature, velocity microorganism and concentration field is graphically evaluated. Moreover, physical quantities are observed via graphs and tabulated data in details. It has been seen by the observation that the involvement of unsteadiness parameter restricts the change of laminar to turbulent flow. Further, for increasing velocity slip parameter velocity component in both directions shows lessening behavior. The Nusselt number exhibits diminishing behavior for larger values of Deborah number, and it shows the opposite behavior for larger values of convective parameter.

## Introduction

To understand the rheological aspect and mechanism, many models have been established for non-Newtonian fluid in the past. The researchers have given a special attention to the nonlinear differential and rate type models. Maxwell model is the rate type model, and they discussed the characteristics of relaxation time. Due to specific application and special stress relaxation properties the non-Newtonian fluid is a talking point for the researchers. Non-Newtonian fluids are detected at chemical and nuclear industries, foodstuffs, bio engineering, polymeric liquids, and material processing. The Maxwell liquid model was proposed by Maxwell^1^ to illustrate the elastic and viscous reaction of air. Zhao et al.^2^ explored the Soret and Dufour impacts with Maxwell MHD liquid in porous surface. Zheng et al.^3^ addressed Maxwell generalized liquid with oscillatory and constantly accelerating sheet. Shateyi^4^ analyzed the MHD flow of Maxwell liquid in the occurrence of chemical reactions and thermophoresis effect on a vertical stretching sheet. Shafiq et al.^5^ deliberated the MHD stagnation point flow of non-Newtonian (Walters-B) fluid across a stretching surface with homogenous-heterogenous reactions and Newtonian heating effect. Farooq et al.^6^ work out the MHD Maxwell fluid flow with nanomaterials through an exponentially extending surface. Rasool et al.^7^ designated the heat and mass transport investigation of Jeffrey MHD nanofluid flow with porous medium over an extending sheet. Khan and Nadeem^8^ propose a comparison of linear and exponential stretching sheets of a rotating Maxwell nanomaterials liquid flow with stratification influence. Some latest research associated to non-Newtonian liquid is given in the Refs.^9–11^.

The transfer of heat is a natural mechanism which happens with temperature differences within the system. Recently, the heat transfer phenomenon as a wave inspired the researchers from all over the world because heat transfer prevalent biomedical and industrial application. For example, electronic device cooling, power generator, heat conduction in tissues, and nuclear reactor cooling etc. The law of energy conduction to the analysis of transport of heat is suggested by Fourier^12^. Cattaneo^13^ modifying the Fourier law to avoid the heat conduction behavior by exerting time relaxation term. Magyari and Keller^14^ presented the mass and heat transport analysis of flow over an exponentially extending surface. The heat transfer of unsteady 3D viscous flow of a boundary layer fluid of the series solution passes an impulsively expanding sheet is employed by Xu et al.^15^. Kuznetsov and Nield^16^ reviewed the characteristics of heat transport in natural convective flow of a Buongiorn’s model through a vertical plate. Gul et al.^17^ presented the characteristic of transport of heat for the 2nd grade time dependent MHD thin film fluid flow analytically using two different ways. Hayat et al.^18^ presented the heat transport analysis of stagnation point MHD flow on a vertical sheet. Gkountas et al.^19^ analyzed the heat transfer of a viscous nanofluid in the presence of various nanoparticles.

The fluid dynamics by a stretching sheet are valuable in extrusion processes. The sheeting material formed in industrial production processes, and they consist of both polymer and metal sheets. The material region between the die and the collecting mechanism may logically assume that the stretching process alter with distance from the die, while cooling begins to stretch because of the solidification that ultimately happen. The current research concentrates to examine the flows by an exponentially stretching sheet. Such flow is quite widespread in applications such as paper production, crystal growing, continuous casting, glass fiber, metallurgical processes etc. The field of geophysical fluid dynamics that naturally occurring on earth is the main application of such fluid motion. The geophysical fluid dynamics contain a larger scale motion on earth, such as oceanography, meteorology, river flow, cloud’s motion etc. The extensibility of the sheet is a valuable aspect of the flow which can be carried out to boost the machinelike feature of the sheet. Flow on an extending sheet first time analyzed by Crane^20^. Later on, Gupta and Gupta^21^ examined the mass and heat transport of liquid flow on an extending surface. Bidin and Nazar^22^ incorporated the two-dimensional viscous liquid in the regime of radiation passes an exponentially extending surface. The flow of viscous liquid passes an exponentially extending surface with MHD is premeditated by Ishak^23^. Liu et al.^24^ conferred the heat transport of 3D viscous fluid flow pass an exponentially surface. Hayat et al.^25^ investigated the transport of heat on stagnant point MHD flow of nanoliquid across the extending surface in the presence of nonlinear radiation. Benos et al.^26^ considered the shrinking / stretching surface to elaborate the transfer of heat on flow of MHD in the existence of radiation. Some studies concerning to stretching surfaces is presented in the Refs.^27–30^.

Bioconvection is a phenomenon that is used to describe the instability and unstructured pattern formed due to the microorganisms, as a result the lesser density particles are swimming to the uppermost portion of a liquid. These complex microorganisms, such as gyrotactic microorganisms like algae, tend to cluster at the upper section of the fluid layer as they swim upwards, resulting in an unstable top heavy density stratification. Moreover, microorganisms are the microscopic organisms that lived everywhere in the surrounding such as deep sea, rocks, equator, deserts etc. The area of oil recovery and geophysical fact the bio-convection has a notable role. Kuznetsov^31^ manifested the oxytactic microorganisms along similarity of finite depth shallow horizontal surface. The micropolar nanofluid with bio-convection recently suggested by Xu and Pop^32^. The mass and heat transport rate of convective flow of Nano liquid on a stretching sheet with microorganism is presented by Shafiq et al.^33^. Nadeem et al.^34^ highlighted the 3D bio-convection nanomaterial liquid flow through an exponentially extending surface with micropolar fluid. Rashed and Nabwey^35^ scrutinized the mixed bioconvection flow of nanomaterial liquid with convective conditions over a circular cylinder. Amer et al.^36^ investigated the dynamical motion of a symmetric rigid body around a principal axis containing the viscous fluid in the existence of gyrostatic moment. Some recent study about gyrotactic microorganisms is found in the Refs.^37–39^.

Motivation of the present work is to examine the three-dimensional bio-convective unsteady Maxwell nanomaterial liquid flow with the convective condition past an exponentially extending surface. The mass and heat transport investigation is represented with the influence of variable thermal conductivity and chemical reaction. The main finding of the current problem is to analyze the convective and concentration boundary condition together on the exponential stretching surface of a Maxwell nanofluid, which in not currently investigated in the literature yet. The transferred equations are tackled by applying bvp4c technique. Graphical outcomes of emerging characteristics are sketched and discussed. Physical behaviors of microorganisms, mass, and heat transport rate are analyzed through graphs and tabulated data.

## Mathematical formulation

**Figure 1:**
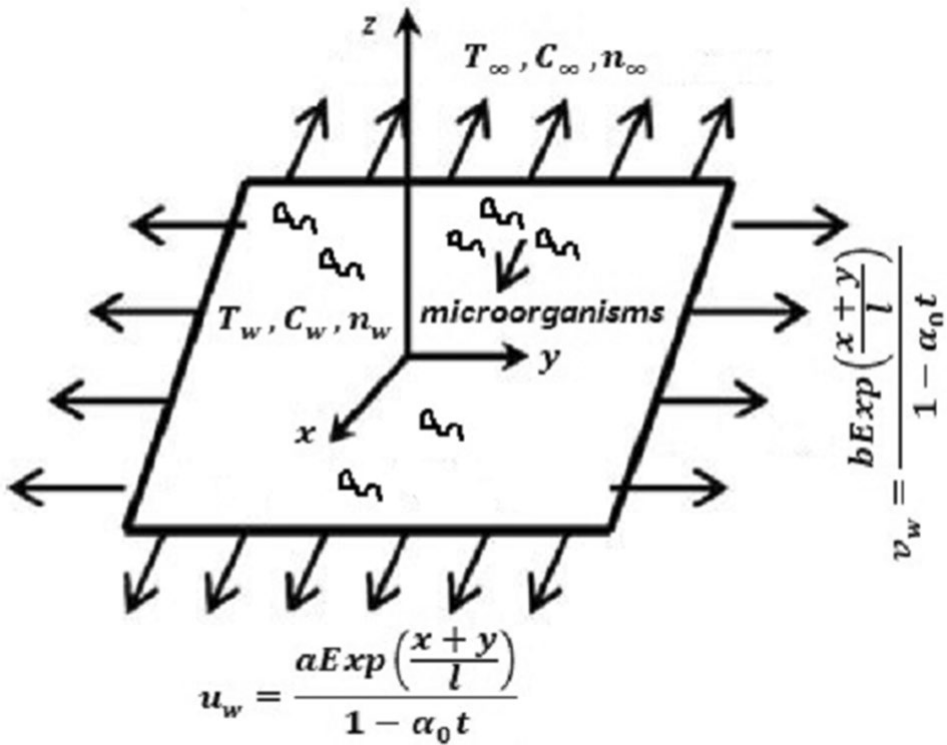
Physical picture of the paper.

We studied an unsteady, incompressible, and three-dimensional flow of chemically reactive Maxwell bio-convective nanomaterials liquid towards an exponentially extending surface with $z = 0.$ The convective and slip boundary condition also taken into account to analyze the mass and heat transport. The flow attends the region $z > 0$ shown in Fig. 1. Let the extending velocities of an exponentially stretching sheet is $u_{w} = \frac{{aExp\left( {\frac{x + y}{l}} \right)}}{{1 - \alpha_{0} t}} and v_{w} = \frac{{bExp\left( {\frac{x + y}{l}} \right)}}{{1 - \alpha_{0} t}}$ in the direction of ${\text{x }}$ and ${\text{y}}$ respectively. Inside the boundary layer $C$, $T$ and $n$ denotes the nanoparticle volume concentration, temperature and microorganism density respectively. Furthermore, nanoparticle volume concentration, temperature and microorganism at the wall is defined by $C_{w} ,T_{w}$ and $n_{w}$ respectively and away from the wall they are $C_{\infty } ,$ $T_{\infty }$ and $n_{\infty }$ respectively. Using above mentioned assumption the flow model takes the following form,

1 $${\mathbf{\nabla }} \cdot {\mathbf{V = }}0,$$

2 $$\rho \frac{{D{\mathbf{V}}}}{Dt} = - {\mathbf{\nabla }} \cdot P + \rho {\mathbf{E}} + {\mathbf{j}} \times {\mathbf{B}} + {\mathbf{\nabla }} \cdot {\mathbf{S}},$$

3 $$\frac{DT}{{Dt}} = \frac{1}{{\rho c_{p} }}{\mathbf{\nabla }} \cdot \left( {k\left( T \right){\mathbf{\nabla }}T} \right) + \tau \left( {\frac{{D_{T} }}{{T_{\infty } }}\nabla T \cdot \nabla T + D_{B} \nabla T \cdot \nabla C} \right),$$

4 $$\frac{DC}{{Dt}} = D_{B} \nabla^{2} C + \frac{{D_{T} }}{{T_{\infty } }}\nabla^{2} T - k_{0} C.$$

5 $$\frac{DN}{{Dt}} = D_{m} \nabla^{2} N + \frac{{\tilde{b}W_{c} }}{\nabla C}\left( {\nabla N \cdot \nabla C} \right).\,$$

Here $D_{T}$ is the thermal diffusivity, $D_{B}$ is the mass diffusivity, $D_{m}$ microorganism diffusivity, $k_{0}$ is the chemical reaction constant, $k\left( T \right)$ is the variable thermal conductivity, $\rho {\mathbf{E + j}} \times {\mathbf{B}}$ is the body forces, $\tilde{b}$ is the chemotaxis constant, $\rho$ is the density of fluid, $W_{c}$ is the cell swimming speed, and $c_{p}$ is specific heat. The ${\mathbf{S}}$ is the extra stress tensor for Maxwell liquid model, which is characterized as,

6 $$\left( {1 + \lambda_{1} \frac{{\mathbf{D}}}{{{\mathbf{Dt}}}}} \right){\mathbf{S = }}\mu {\mathbf{A}}_{1} ,$$

where ${\mathbf{A}}_{1}$ is the Rivlin-Ericksen tensor $\left( {{\mathbf{A}}_{1} = \left( {\nabla {\mathbf{V}}} \right)^{t} + \nabla {\mathbf{V}}} \right)$, $\mu$ is the viscosity, $\lambda_{1}$ is the relaxation time, and $\frac{{\mathbf{D}}}{{{\mathbf{Dt}}}}$ is the material derivative. The governing equations of mass, momentum, energy, concentration, and microorganism by using the boundary layer approximation and Eqs. (1–6) takes the following form^40^,

7 $$\frac{\partial u}{{\partial x}} + \frac{\partial v}{{\partial y}} + \frac{\partial w}{{\partial z}} = 0,$$

8 $$\begin{aligned} \frac{\partial u}{{\partial t}} & + u\frac{\partial u}{{\partial x}} + v\frac{\partial u}{{\partial y}} + w\frac{\partial u}{{\partial z}} + \lambda_{1} \left( {\frac{{\partial^{2} u}}{{\partial t^{2} }} + 2u\frac{{\partial^{2} u}}{\partial x\partial t} + 2v\frac{{\partial^{2} u}}{\partial y\partial t}} \right) + \frac{{\sigma B^{2} }}{\rho }\left( {u + \lambda_{1} \left( {\frac{\partial u}{{\partial t}} + w\frac{\partial u}{{\partial z}}} \right)} \right) \\ & + \lambda_{1} \left( {\begin{array}{*{20}c} {2w\frac{{\partial^{2} u}}{\partial z\partial t} + u^{2} \frac{{\partial^{2} u}}{{\partial x^{2} }}} \\ { + v^{2} \frac{{\partial^{2} u}}{{\partial y^{2} }} + w^{2} \frac{{\partial^{2} u}}{{\partial z^{2} }}} \\ \end{array} } \right) + \lambda_{1} \left( {\begin{array}{*{20}c} {2uv\frac{{\partial^{2} u}}{\partial x\partial y} + 2vw\frac{{\partial^{2} u}}{\partial y\partial z}} \\ { + 2uw\frac{{\partial^{2} u}}{\partial x\partial z}} \\ \end{array} } \right) = \frac{\mu }{\rho }\left( {\frac{{\partial^{2} u}}{{\partial z^{2} }}} \right), \\ \end{aligned}$$

9 $$\begin{aligned} \frac{\partial v}{{\partial t}} & + u\frac{\partial v}{{\partial x}} + v\frac{\partial v}{{\partial y}} + w\frac{\partial v}{{\partial z}} + \lambda_{1} \left( {\frac{{\partial^{2} v}}{{\partial t^{2} }} + 2u\frac{{\partial^{2} v}}{\partial x\partial t} + 2v\frac{{\partial^{2} v}}{\partial y\partial t}} \right) + \frac{{\sigma B^{2} }}{\rho }\left( {v + \lambda_{1} \left( {\frac{\partial v}{{\partial t}} + w\frac{\partial v}{{\partial z}}} \right)} \right) \\ & + \lambda_{1} \left( {\begin{array}{*{20}c} {2w\frac{{\partial^{2} v}}{\partial z\partial t} + u^{2} \frac{{\partial^{2} v}}{{\partial x^{2} }} + } \\ {v^{2} \frac{{\partial^{2} v}}{{\partial y^{2} }} + w^{2} \frac{{\partial^{2} v}}{{\partial z^{2} }}} \\ \end{array} } \right) + \lambda_{1} \left( {\begin{array}{*{20}c} {2uv\frac{{\partial^{2} v}}{\partial x\partial y} + 2vw\frac{{\partial^{2} v}}{\partial y\partial z}} \\ { + 2uw\frac{{\partial^{2} v}}{\partial x\partial z}} \\ \end{array} } \right) = \frac{\mu }{\rho }\left( {\frac{{\partial^{2} v}}{{\partial z^{2} }}} \right), \\ \end{aligned}$$

10 $$\begin{aligned} \frac{\partial T}{{\partial t}} & + u\frac{\partial T}{{\partial x}} + v\frac{\partial T}{{\partial y}} + w\frac{\partial T}{{\partial z}} = \frac{1}{{\rho c_{p} }}\frac{\partial }{\partial z}\left( {k\left( T \right)\frac{\partial T}{{\partial z}}} \right) \\ & + \tau D_{B} \frac{\partial T}{{\partial z}}\frac{\partial C}{{\partial z}} + \frac{{\tau D_{T} }}{{T_{\infty } }}\left( {\frac{\partial T}{{\partial z}}} \right)^{2} + \frac{{\sigma B^{2} }}{{\rho C_{p} }}\left( {u^{2} + v^{2} } \right) + \frac{\mu }{{\rho C_{p} }}\left( {\left( {\frac{\partial u}{{\partial z}}} \right)^{2} + \left( {\frac{\partial v}{{\partial z}}} \right)^{2} } \right), \\ \end{aligned}$$

11 $$\frac{\partial C}{{\partial t}} + u\frac{\partial C}{{\partial x}} + v\frac{\partial C}{{\partial y}} + w\frac{\partial C}{{\partial z}} = D_{B} \left( {\frac{{\partial^{2} C}}{{\partial z^{2} }}} \right) + \frac{{D_{T} }}{{T_{\infty } }}\frac{{\partial^{2} T}}{{\partial z^{2} }} - k_{0} \left( {C - C_{\infty } } \right),$$

12 $$\frac{\partial n}{{\partial t}} + u\frac{\partial n}{{\partial x}} + v\frac{\partial n}{{\partial y}} + w\frac{\partial n}{{\partial z}} = D_{m} \left( {\frac{{\partial^{2} n}}{{\partial z^{2} }}} \right) - \frac{{\tilde{b}W_{c} }}{{C_{w} - C_{\infty } }}\frac{\partial }{\partial z}\left( {n\frac{\partial C}{{\partial z}}} \right).$$

The related boundary conditions are^41^,

13 $$\left( {\begin{array}{*{20}c} {u - u_{w} = 0, v - v_{w} = 0, w = 0} \\ {\begin{array}{*{20}c} {k\left( T \right)\frac{\partial T}{{\partial z}} + h_{w} \left( {T_{w} - T} \right) = 0, C_{w} + H\frac{\partial C}{{\partial z}} = 0, n = n_{w} . at z \to 0, } \\ {u = 0, v = 0, T \to T_{\infty } , C \to C_{\infty } , n \to n_{\infty } at z \to \infty .} \\ \end{array} } \\ \end{array} } \right)$$

The velocity components in $x, y,$ and $z$ directions are $u, v,$ and $w$ respectively. The symbols $H$ is the concentration slip factor, $\tau$ is the ratio between heat capacity of nanoparticles to the base fluid, and $h_{w}$ illustrates the heat transport coefficient. Furthermore, ${ }k\left( T \right) = k_{\infty } \left( {1 + raj\theta } \right)$^42^ is signified the variable thermal conductivity in which $k_{\infty }$ indicates the thermal conductivity of the surrounding.

To transform the flow model PDEs into non-dimensionalized form, we introduced the following non dimensional variable^43^,

14 $$\begin{aligned} \eta & = \sqrt {\frac{a}{{2\nu l\left( {1 - \alpha_{0} t} \right)}}} zExp\left( {\frac{x + y}{{2l}}} \right), u = \frac{ax}{{\left( {1 - \alpha_{0} t} \right)}}Exp\left( {\frac{x + y}{l}} \right)f^{^{\prime}} \left( \eta \right),v = \frac{ax}{{\left( {1 - \alpha_{0} t} \right)}}Exp\left( {\frac{x + y}{l}} \right)g^{\prime}\left( \eta \right), \\ w & = - \sqrt {\frac{\nu a}{{2l\left( {1 - \alpha_{0} t} \right)}}} Exp\left( {\frac{x + y}{{2l}}} \right)\left( {f\left( \eta \right) + g\left( \eta \right) + \eta \left( {f^{\prime}\left( \eta \right) + g^{\prime}\left( \eta \right)} \right)} \right), \\ T & = T_{\infty } + \frac{{T_{0} Exp\left( {\frac{x + y}{{2l}}} \right)}}{{\left( {1 - \alpha_{0} t} \right)^{2} }} \theta \left( \eta \right),\,T = C_{\infty } + \frac{{C_{0} Exp\left( {\frac{x + y}{{2l}}} \right)}}{{\left( {1 - \alpha_{0} t} \right)^{2} }}\phi \left( \eta \right),\,n = n_{\infty } + \frac{{n_{0} Exp\left( {\frac{x + y}{{2l}}} \right)}}{{\left( {1 - \alpha_{0} t} \right)^{2} }}\chi \left( \eta \right), \\ \theta \left( \eta \right) & = \frac{{T - T_{\infty } }}{{T_{w} - T_{\infty } }}, \phi \left( \eta \right) = \frac{{C - C_{\infty } }}{{C_{w} - C_{\infty } }},\,\chi \left( \eta \right) = \frac{{n - n_{\infty } }}{{n_{w} - n_{\infty } }}. \\ \end{aligned}$$

Here $T_{0}$, $C_{0}$, and $n_{0}$ all are the constants. Using Eq. (14), the equation of continuity automatically holds, and other Eqs. (8–12) take the following form,

15 $$\begin{aligned} f^{\prime\prime\prime} & - 2f^{^{\prime}} \left( {f^{\prime} + g^{\prime}} \right) + f^{\prime\prime}\left( {f + g} \right) - A\left[ {2f^{^{\prime}} + \eta f^{\prime\prime}} \right] - \beta \left[ {A^{2} \left( {4f^{\prime} + \frac{7\eta }{2}f^{\prime\prime} + \frac{{\eta^{2} }}{2}f^{\prime\prime\prime}} \right)} \right] \\ & - \beta \left[ {\begin{array}{*{20}c} {A\left\{ {\left( {4f^{\prime} + 2\eta f^{\prime\prime}} \right)\left( {f^{\prime} + g^{\prime}} \right) - \left( {3f^{\prime\prime} + \eta f^{\prime\prime\prime}} \right)\left( {f + g} \right)} \right\}} \\ { + \left( {2f^{\prime} - \frac{\eta }{2}f^{\prime\prime}} \right)\left( {f^{\prime} + g^{\prime}} \right)^{2} - 3f^{\prime\prime}\left( {f + g} \right)\left( {f^{\prime} + g^{\prime}} \right) + \frac{{\left( {f + g} \right)^{2} }}{2}f^{\prime\prime\prime}} \\ \end{array} } \right] \\ & - M\left[ {f^{\prime} + \beta \left( {f + g} \right)f^{\prime\prime} + \frac{\beta A}{2}\eta f^{\prime\prime} + \beta Af^{\prime}} \right] = 0, \\ \end{aligned}$$

16 $$\begin{aligned} g^{\prime\prime\prime} & - 2g^{^{\prime}} \left( {f^{\prime} + g^{\prime}} \right) + g^{\prime\prime}\left( {f + g} \right) - A\left[ {2g^{^{\prime}} + \eta g^{\prime\prime}} \right] - \beta \left[ {A^{2} \left( {4g^{\prime} + \frac{7\eta }{2}g^{\prime\prime} + \frac{{\eta^{2} }}{2}g^{\prime\prime\prime}} \right)} \right] \\ & - \beta \left[ {\begin{array}{*{20}c} {A\left\{ {\left( {4g^{\prime} + 2\eta g^{\prime\prime}} \right)\left( {f^{\prime} + g^{\prime}} \right) - \left( {3g^{\prime\prime} + \eta g^{\prime\prime\prime}} \right)\left( {f + g} \right)} \right\}} \\ { + \left( {2g^{\prime} - \frac{\eta }{2}g^{\prime\prime}} \right)\left( {f^{\prime} + g^{\prime}} \right)^{2} - 3g^{\prime\prime}\left( {f + g} \right)\left( {f^{\prime} + g^{\prime}} \right) + \frac{{\left( {f + g} \right)^{2} }}{2}g^{\prime\prime\prime}} \\ \end{array} } \right] \\ & - M\left[ {g^{\prime} + \beta \left( {f + g} \right)g^{\prime\prime} + \frac{\beta A}{2}\eta g^{\prime\prime} + \beta Ag^{\prime}} \right] = 0, \\ \end{aligned}$$

17 $$\left( {1 + \theta } \right)\theta^{\prime\prime} + raj\theta^{{\prime}{2}} - Pr\left[ \begin{array}{*{20}c} {\left( {f^{\prime}+ g^{\prime}} \right)\theta + \left( {f + g} \right)\theta^{\prime}- A\left( {4\theta + \eta \theta^{\prime}} \right)} \\ + Nb\theta^{\prime}\phi^{\prime} + Nt\theta^{{\prime}{2}} - M\left({Ec_{1} f^{{\prime}{2}} + Ec_{2} g^{{\prime}{2}} } \right) \\ { -\left( {Ec_{1} f^{\prime\prime 2} + Ec_{2}g^{\prime\prime 2} } \right)} \\ \end{array} \right] = 0,$$

18 $$\phi^{\prime\prime} - Sc\left[ {\left( {f^{\prime} + g^{\prime}} \right)\phi + \left( {f + g} \right)\phi^{\prime} - A\left( {4\phi + \eta \phi^{\prime}} \right) + \sigma \phi } \right] + \frac{Nt}{{Nb}}\theta^{\prime\prime} = 0,$$

19 $$\chi^{\prime\prime} - Sb\left[ {\left( {f^{\prime} + g^{\prime}} \right)\chi + \left( {f + g} \right)\chi^{\prime} - A\left( {4\chi + \eta \chi^{\prime}} \right)} \right] - Pe\left( {\left( {\chi + \pi } \right)\phi^{\prime\prime} + \chi ^{\prime}\phi^{\prime}} \right) = 0.$$

The dimensionless form of the boundary conditions is,

20 $$\begin{aligned} f\left( \eta \right) + g\left( \eta \right) & = 0,\, f^{^{\prime}} \left( \eta \right) = 1,\, g^{\prime}\left( \eta \right) = \lambda , \\ \left( {1 + raj\theta \left( \eta \right)} \right)\theta^{\prime}\left( \eta \right) & = - \gamma \left( {1 - \theta \left( \eta \right)} \right), \phi \left( \eta \right) = 1 + \delta_{1} \phi ^{\prime}\left( \eta \right) = 0 \,{\text{at}}\,\eta \to 0, \\ f^{\prime}\left( \eta \right) & = g^{\prime}\left( \eta \right) = \theta \left( \eta \right) = \phi \left( \eta \right) = 0\,{\text{at}}\,\eta \to \infty . \\ \end{aligned}$$

Here prime stand for derivative with respect to $\eta$. The symbols $A$, $\beta ,$ $Pr,$ $Sc,$ $\lambda ,$ $\delta_{1}$,$raj$ and $\gamma$ are represented the unsteadiness parameter, Deborah number, Prandtl number, Schmidt number, stretching ratio characteristic, concentration slip parameter, thermal conductivity parameter and convection characteristic respectively. The symbols $Nb$, $Pe$, $Nt$ and $Sb$ denotes Brownian motion parameter, Peclet number, the thermophoresis parameter and bio-convection Schmidt number respectively. These parameters are defined as,

21 $$\begin{aligned} A & = \frac{{l\alpha_{0} }}{a}, \beta = \frac{{\lambda_{1} a}}{{2l\left( {1 - \alpha_{0} t} \right)}}, Pr = \frac{{c_{p} \mu }}{{k_{\infty } }}, \,Sc = \frac{\nu }{{D_{B} }}, \lambda = \frac{b}{a}, \gamma = - \frac{{h_{w} }}{{k_{\infty } }} \sqrt {\frac{2\nu l}{{a\left( {1 - \alpha_{0} t} \right)}}} , \\ Pe & = \frac{{\tilde{b}D_{m} W_{c} }}{{D_{B} }},\,\delta_{1} = H\sqrt {\frac{\alpha }{{2\nu l\left( {1 - \alpha_{0} t} \right)}}} ,\,Sb = \frac{\nu }{{D_{m} }}, Nt = \frac{{\tau {\Delta }TD_{T} }}{{\nu T_{\infty } }}, Nb = \frac{{\tau {\Delta }CD_{B} }}{\nu }. \\ \end{aligned}$$

Here we take $Exp\left( {\frac{x + y}{{2l}}} \right) = 1 + o\left( 1 \right).$

### Physical quantities

In terms of engineering, the physical quantities are the most important. These physical quantities observed the mass, heat, and microorganism transport rate. These quantities are defined as,

22 $$Nu_{x} = \frac{{xq_{m} }}{{k\left( T \right)\left( {T_{w} - T_{\infty } } \right)}} , Sh_{x} = \frac{{xj_{w} }}{{D_{B} \left( {C_{w} - C_{\infty } } \right)}}, Q_{nx} = \frac{{xz_{w} }}{{D_{m} n_{w} }}.$$

In Eq. (22) $q_{m}$,$z_{w} {\text{and}}$ $j_{w}$ shows the heat, microorganism and mass fluxes, respectively. These are given as,

23 $$\left. {q_{m} = - k\left( T \right)\left( {\frac{\partial T}{{\partial z}}} \right)} \right|_{z \to 0} , \left. {j_{w} = - D_{B} \left( {\frac{\partial C}{{\partial z}}} \right)} \right|_{z \to 0} , \left. {z_{w} = - D_{m} \left( {\frac{\partial n}{{\partial z}}} \right)} \right|_{z \to 0} .$$

The physical quantities in the dimensionless from are,

24 $$\begin{aligned} Nu_{x} \left( {Re_{x} } \right)^{ - 0.5} & = - \theta^{\prime}\left( 0 \right), \\ Sh_{x} \left( {Re_{x} } \right)^{ - 0.5} & = - \phi^{\prime}\left( 0 \right), \\ Qn_{x} \left( {Re_{x} } \right)^{ - 0.5} & = - \chi^{\prime}\left( 0 \right). \\ \end{aligned}$$

The local Reynolds number is $Re_{x} = u_{w} \sqrt {\frac{{2l\left( {1 - \alpha_{0} t} \right)}}{\nu a}}$.

### Solution procedure

The nonlinear system of Eqs. (15–19) with Eq. (20) are solved numerically with the help of bvp4c MATLAB solution technique. To use a numerical method first we convert the Eqs. (15–20) into the system of first order differential equations. The convergence criteria were assigned as 10^−6^ ^44,45^. Table 1 demonstrated the comparison of a temperature gradient with the previously published result of Nadeem et al.^44^.

**Table 1:** Previous studies comparison of $\theta ^{\prime}(0)$ values, when $M = \gamma = 0 = \varepsilon$.

	Nadeem et al.^44^	Presents results
$\Pr$	$\theta ^{\prime}(0)$	$\theta ^{\prime}(0)$
**0.72**	0.809400	0.809401
**1.0**	1.000000	1.000000
**3.0**	1.923682	1.923683
**10.0**	3.720673	3.720674
**100.0**	12.29408	12.29409

The system of equations of first order,

25 $$\left( {\begin{array}{*{20}c} {y\left( 1 \right) = f, y\left( 2 \right) = f^{^{\prime}} , y\left( 3 \right) = f^{^{\prime\prime}} } \\ {y\left( 4 \right) = g, y\left( 5 \right) = g^{^{\prime}} , y\left( 6 \right) = g^{\prime\prime}} \\ \end{array} } \right),$$

26 $$yy_{1} = \left[ {\begin{array}{*{20}c} {1 - \beta A^{2} \frac{\eta }{2}^{2} } \\ { + \beta A\eta \left\{ {y\left( 1 \right) + y\left( 4 \right)} \right\}} \\ { - \beta \frac{{\left( {y\left( 1 \right) + y\left( 4 \right)} \right)^{2} }}{2}} \\ \end{array} } \right]^{ - 1} \left[ {\begin{array}{*{20}c} {A\left( {2y\left( 2 \right) + \eta y\left( 3 \right)} \right) + 2y\left( 2 \right)y\left( 2 \right) + 2y\left( 2 \right)y\left( 5 \right)} \\ { - 3\beta \left( {y\left( 2 \right) + y\left( 5 \right)} \right)\left( {y\left( 1 \right) + y\left( 4 \right)} \right)y\left( 3 \right)} \\ { + \beta A\left\{ {\left( {y\left( 2 \right) + y\left( 5 \right)} \right)\left( {2\eta y\left( 3 \right) + 4y\left( 2 \right)} \right)} \right\} - y\left( 1 \right)y\left( 3 \right)} \\ { - \beta \left( {y\left( 5 \right) + y\left( 2 \right)} \right)^{2} \left( {\frac{\eta }{2}y\left( 3 \right) - 2y\left( 2 \right)} \right) - y\left( 4 \right)y\left( 3 \right)} \\ { + \beta A^{2} \left( {\frac{7\eta }{2}y\left( 3 \right) + 4y\left( 2 \right)} \right) - 3\beta A\left( {y\left( 1 \right)y\left( 3 \right) + y\left( 3 \right)y\left( 4 \right)} \right)} \\ { + M\left[ {y\left( 2 \right) + \beta \left( {y\left( 1 \right) + y\left( 4 \right)} \right)y\left( 3 \right) + \frac{\beta A}{2}\eta y\left( 3 \right) + \beta Ay\left( 2 \right)} \right]} \\ \end{array} } \right],$$

27 $$yy_{2} = \left[ {\begin{array}{*{20}c} {1 - \beta A^{2} \frac{\eta }{2}^{2} } \\ { + \beta A\eta \left\{ {y\left( 1 \right) + y\left( 4 \right)} \right\}} \\ { - \beta \frac{{\left( {y\left( 1 \right) + y\left( 4 \right)} \right)^{2} }}{2}} \\ \end{array} } \right]^{ - 1} \left[ {\begin{array}{*{20}c} {A\left( {2y\left( 5 \right) + \eta y\left( 6 \right)} \right) + 2y\left( 2 \right)y\left( 5 \right) + 2y\left( 5 \right)y\left( 5 \right)} \\ { - 3\beta y\left( 6 \right)\left( {y\left( 1 \right) + y\left( 4 \right)} \right)\left( {y\left( 2 \right) + y\left( 5 \right)} \right)} \\ { + \beta A\left\{ {\left( {y\left( 2 \right) + y\left( 5 \right)} \right)\left( {2\eta y\left( 6 \right) + 4y\left( 5 \right)} \right)} \right\} - y\left( 1 \right)y\left( 6 \right)} \\ { - \beta \left( {y\left( 5 \right) + y\left( 2 \right)} \right)^{2} \left( {\frac{\eta }{2}y\left( 6 \right) + 2y\left( 5 \right)} \right) - y\left( 4 \right)y\left( 6 \right)} \\ { + \beta A^{2} \left( {\frac{7\eta }{2}y\left( 6 \right) + 4y\left( 5 \right)} \right) - 3\beta A\left( {y\left( 1 \right)y\left( 6 \right) + y\left( 6 \right)y\left( 4 \right)} \right)} \\ { + M\left[ {y\left( 5 \right) + \beta \left( {y\left( 1 \right) + y\left( 4 \right)} \right)y\left( 6 \right) + \frac{\beta A}{2}\eta y\left( 6 \right) + \beta Ay\left( 5 \right)} \right]} \\ \end{array} } \right],$$

28 $$\left( {\begin{array}{*{20}c} {\theta = y\left( 7 \right), \theta^{^{\prime}} = y\left( 8 \right), \phi = y\left( 9 \right)} \\ { \phi^{^{\prime}} = y\left( {10} \right), \chi = y\left( {11} \right), \chi^{^{\prime}} = y\left( {12} \right)} \\ \end{array} } \right),$$

29 $$yy_{3} = \left[ {\begin{array}{*{20}c} {1 + rajy\left( 7 \right)} \\ \end{array} } \right]^{ - 1} \left[ {\begin{array}{*{20}c} {Pr\left( {y\left( 2 \right) + y\left( 5 \right)} \right)y\left( 7 \right) - Pr\left( {y\left( 1 \right) + y\left( 4 \right)} \right)y\left( 8 \right) - y\left( 8 \right)^{2} } \\ { + Pr\left( {A\left\{ {\eta y\left( 8 \right) + 4y\left( 7 \right)} \right\} - Nby\left( 8 \right)y\left( {10} \right) - Nty\left( 8 \right)y\left( 8 \right)} \right)} \\ { + M\left( {Ec_{1} y\left( 2 \right)^{2} + Ec_{2} y\left( 5 \right)^{2} } \right) + \left( {Ec_{1} y\left( 3 \right)y\left( 3 \right) + Ec_{2} y\left( 6 \right)y\left( 6 \right)} \right)} \\ \end{array} } \right],$$

30 $$yy_{4} = Sc\left[ {\begin{array}{*{20}c} {y\left( 9 \right)\left( {y\left( 2 \right) + y\left( 5 \right)} \right) + A\left\{ {\eta y\left( {10} \right) + 4y\left( 9 \right)} \right\}} \\ { - y\left( {10} \right)\left( {y\left( 1 \right) + y\left( 4 \right)} \right)} \\ \end{array} } \right] - \frac{Nt}{{Nb}}\,yy_{3} ,$$

31 $$yy_{5} = Sb\left[ {\begin{array}{*{20}c} {\left( {y\left( 2 \right) + y\left( 5 \right)} \right)y\left( {11} \right)} \\ { + A\left\{ {\eta y\left( {12} \right) + 4y\left( {11} \right)} \right\}} \\ { - \left( {y\left( 1 \right) + y\left( 4 \right)} \right)y\left( {12} \right)} \\ \end{array} } \right] + Pe y\left( {10} \right)y\left( {12} \right) + Pe \left( {y\left( {11} \right) + \pi } \right)yy_{4} .$$

The appropriative conditions are,

32 $$\begin{aligned} y_{0} \left( 1 \right) & = 0, y_{0} \left( 2 \right) = 1, y_{0} \left( 4 \right) = 0, y_{0} \left( 5 \right) = \lambda , \\ \left( {1 + rajy_{0} \left( 7 \right)} \right)y_{0} \left( 8 \right) & = - \gamma \left( {1 - y_{0} \left( 7 \right)} \right), y_{0} \left( 9 \right) = 1 + \delta_{1} y_{0} \left( {10} \right) = 0, y_{0} \left( {11} \right) = 1. \\ y_{inf} \left( 2 \right) & = y_{inf} \left( 5 \right) = y_{inf} \left( 7 \right) = y_{inf} \left( 9 \right) = y_{inf} \left( {11} \right) = 0. \\ \end{aligned}$$

## Physical description

The central aim of this work is to determine the Maxwell bio-convective nanomaterial liquid flow on an exponentially extending surface subject to the convective condition. Equations (9)–(14) are numerically manipulated by using bvp4c MATLAB technique. Further, graphically conclusions are conducted for different characteristic on the concentration, velocity, temperature and microorganism profile respectively. The parameter values is specified in the range of $A$ (0.0–0.5), $\lambda$(0.0–1.0), $\beta$(0.0–1.0), $Pr$(1.0–3.0), $Nt$(0.1–1.5), $Nb$(0.1–1.0), $Pe$(1.0–4.0), $Sb$(2.0–4.0), $\gamma$(0.0–1.0), $raj$(0.0–3.0), $\delta_{1}$(0.0–1.5) and $S_{c}$(1.0–4.0). In Table 2 the variation of physical quantities like Nusselt number, microorganism number and Sherwood number are observed. It is examined that the heat and microorganism transfer rate depict flourishing behavior by the boosting values of unsteadiness parameter $\left( A \right)$ and stretching parameter $\left( \lambda \right)$, but the mass transfer rate shows reverse trend. Further, when growing the amount of the Nusselt number, Deborah number $\left( \beta \right)$ and microorganism number displays lessening behavior, while the Sherwood number shows enlarging trend for $\beta$. The tabulated data demonstrates that for larger amount of $Pr$, the microorganism number and Sherwood number are decline, but heat transfer rate rises. Further for greater values of Brownian motion parameter $\left( {Nt} \right)$ both microorganism number and Sherwood number enhances, while the Nusselt number reduces for $Nt$. It is clarified from Table 2 that, microorganism number and Sherwood number displays decreasing behavior for distinct values of thermophoresis characteristic $\left( {Nb} \right)$. Moreover, it is demonstrated that different amount of Peclet number $\left( {Pe} \right)$ the tabulated data represents the reduction behavior for microorganism number.

**Table 2:** Table of $Nu_{x} \left( {Re_{x} } \right)^{ - 0.5}$, $Sh_{x} \left( {Re_{x} } \right)^{ - 0.5}$, and $Qn_{x} \left( {Re_{x} } \right)^{ - 0.5}$ for different parameters when $\gamma = \varepsilon = 0.3.$

$A$	$\lambda$	$\beta$	$Pr$	$Nt$	$Nb$	$Pe$	$- \theta ^{\prime}\left( 0 \right)$	$- \phi ^{\prime}\left( 0 \right)$	$- \chi ^{\prime}\left( 0 \right)$
0.0	0.5	0.3	2.5	0.3	0.3	1.5	0.3722	0.2898	1.600
0.2	–	–	–	–	–	–	0.4124	0.2327	1.8440
0.3	–	–	–	–	–	–	0.4249	0.2155	1.9620
0.3	0.5	0.3	–	0.3	–	–	0.4249	0.2155	1.9620
–	0.7	–	2.5	–	0.3	1.5	0.4279	0.2114	1.9970
–	0.9	–	–	–	–	–	0.4307	0.2076	2.030
0.3	0.5	0.0	–	0.3	–	–	0.4276	0.2119	1.9930
–	–	0.3	2.5	–	–	1.5	0.4249	0.2155	1.9620
0.3	0.5	0.5	–	–	0.3	–	0.4235	0.2174	1.9460
–	–	0.3	2.0	0.3	–	–	0.4119	0.2334	2.004
0.3	–	–	3.0	–	0.3	–	0.4350	0.2019	1.9280
–	–	–	4.0	–	–	1.5	0.4498	0.1820	1.8770
–	0.5	0.3	2.5	0.2	–	–	0.4283	0.1406	1.8620
0.3	–	–	–	0.4	–	–	0.4215	0.2936	2.0740
–	–	–	–	0.6	–	1.5	0.4145	0.4595	2.3340
–	0.5	0.3	–	0.3	0.1	–		0.6466	2.5910
0.3	–	–	2.5	–	0.3	–		0.2155	1.9620
–	–	–	–	–	0.5	–		0.1293	1.8520
–	–	–	–	–	0.3	1.0			1.8700
–	–	–	–	0.3	–	1.5			1.9620
0.2	0.1	0.3	0.3	0.5	0.0	2.0			2.0580

**Figure 2:**
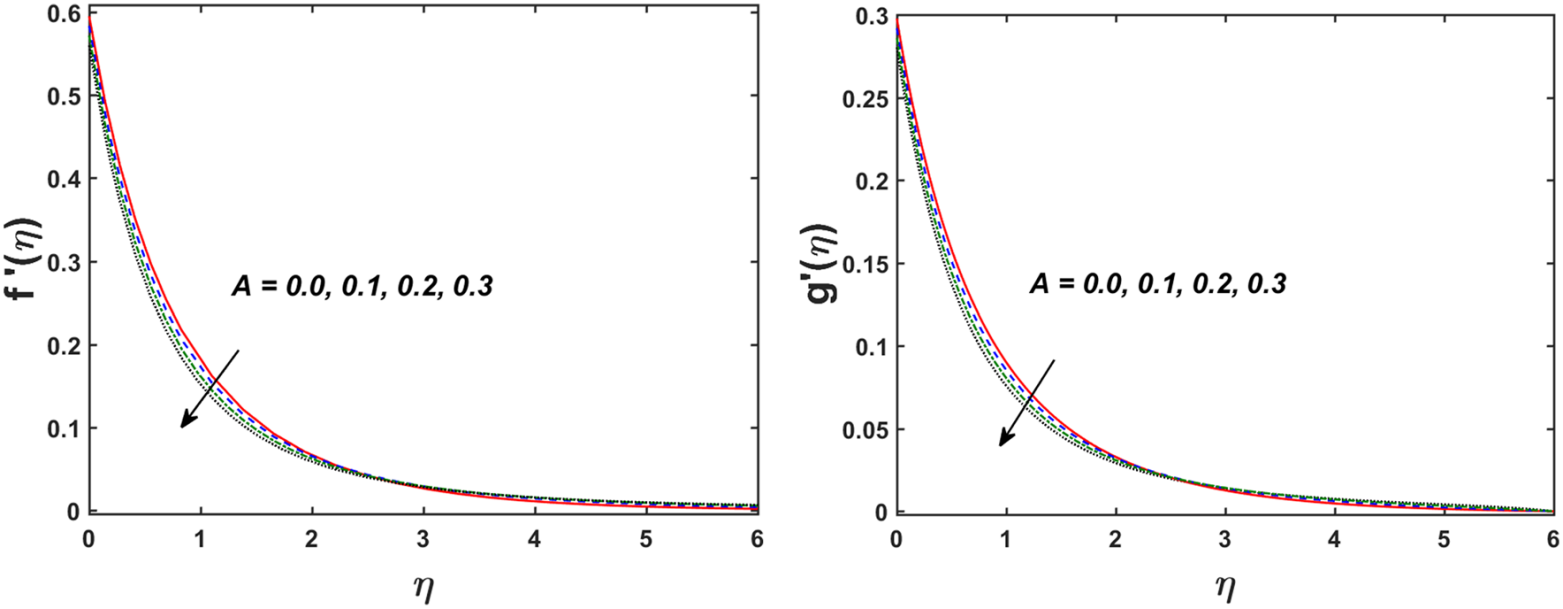
Variation in $f^{\prime}\left( \eta \right)$ and $g^{\prime}\left( \eta \right)$ for $A$.

**Figure 3:**
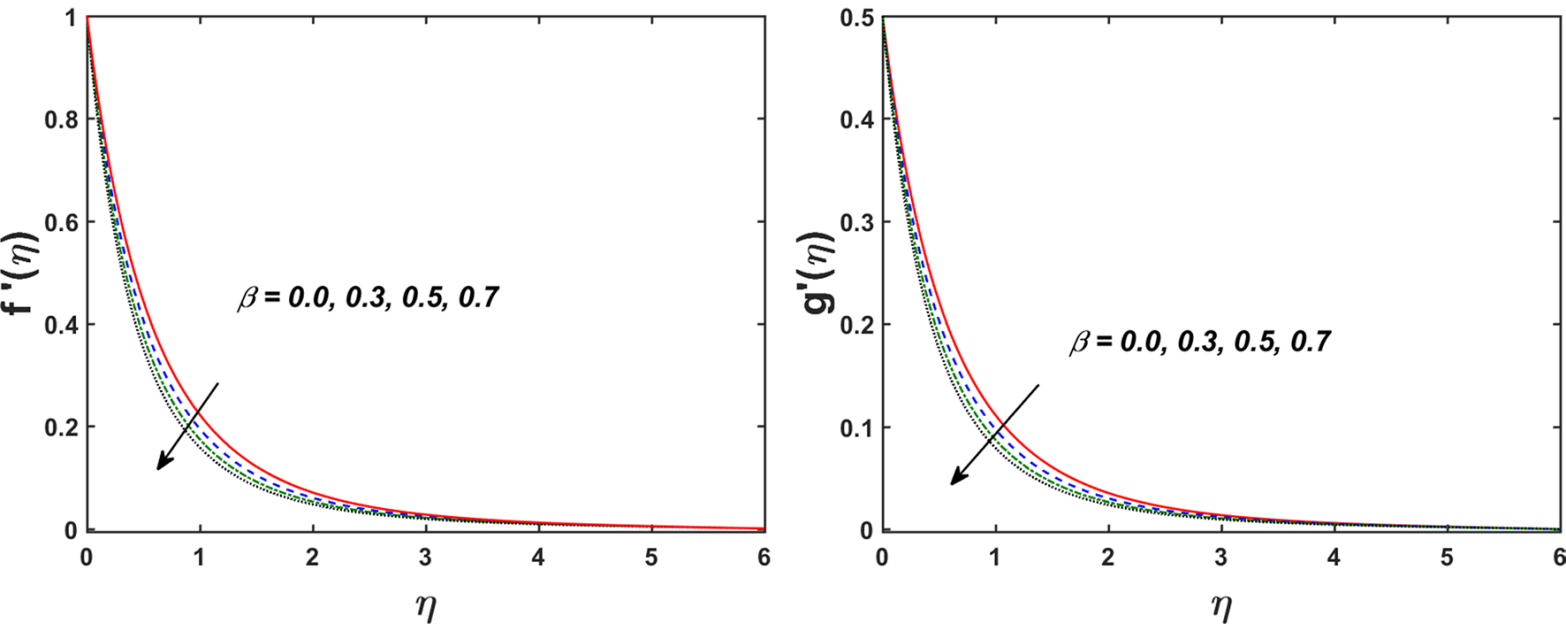
Variation in $f^{\prime}\left( \eta \right)$ and $g^{\prime}\left( \eta \right)$ for $\beta$.

**Figure 4:**
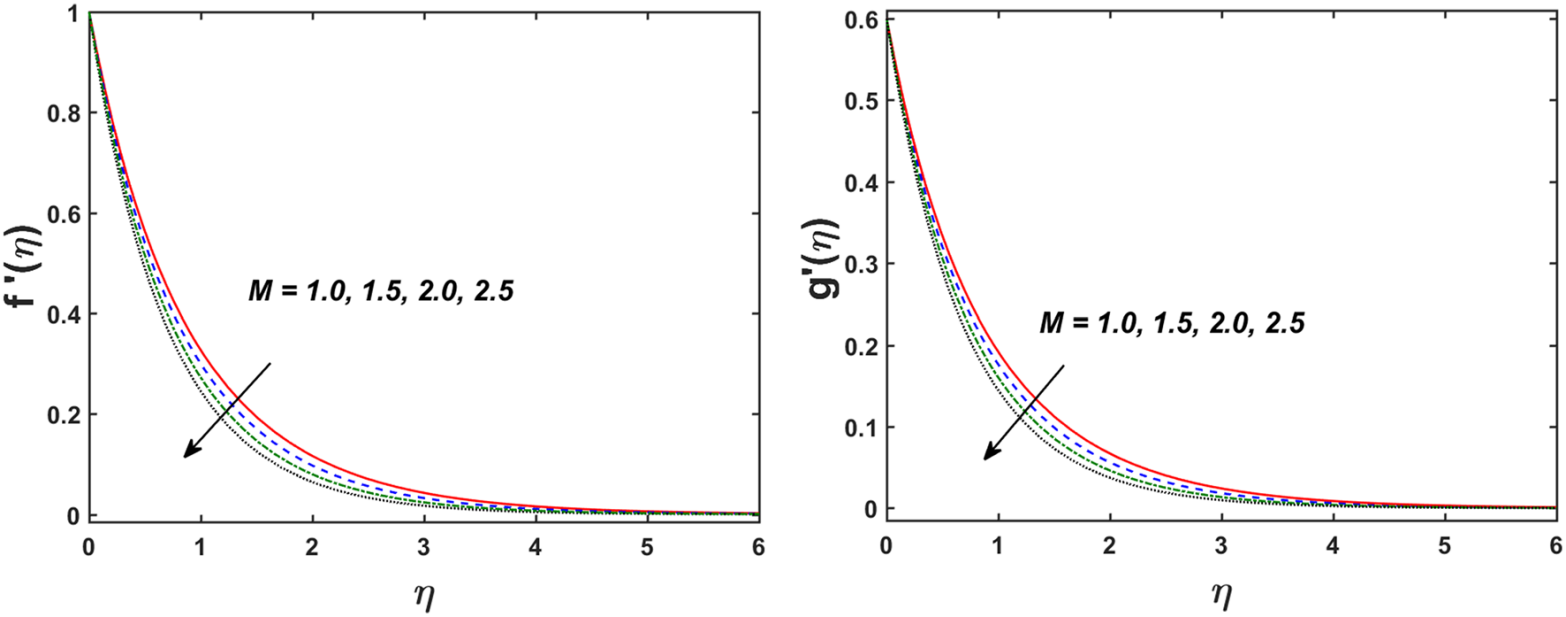
Variation in $f^{\prime}\left( \eta \right)$ and $g^{\prime}\left( \eta \right)$ for $M$.

**Figure 5:**
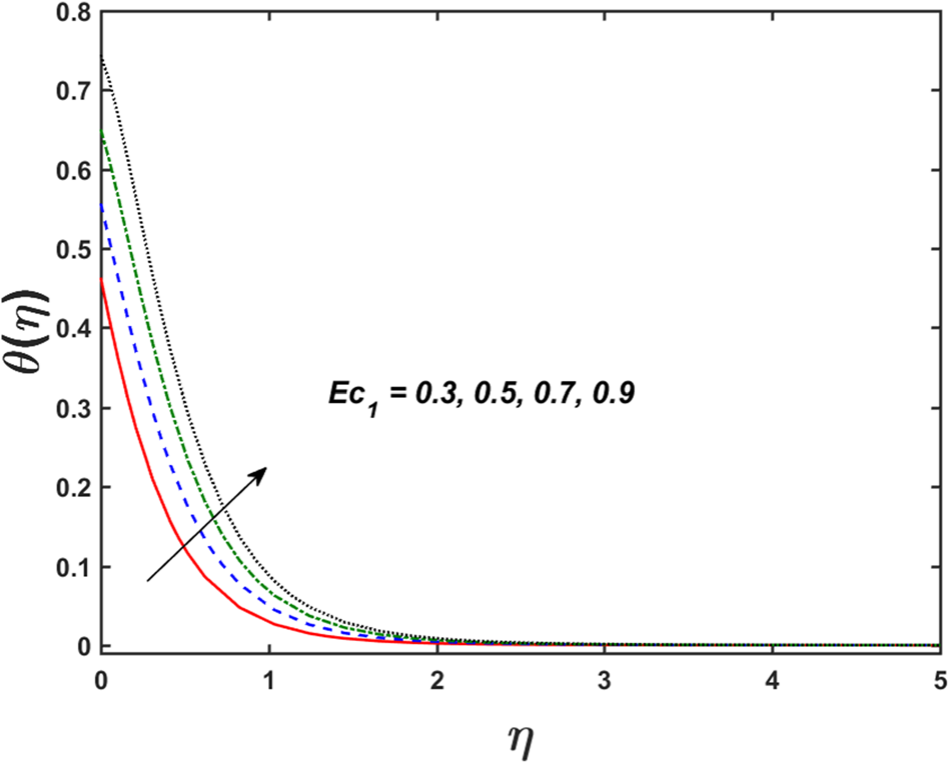
Variation in $\theta \left( \eta \right)$ for $M$ and $Ec_{1}$.

**Figure 6:**
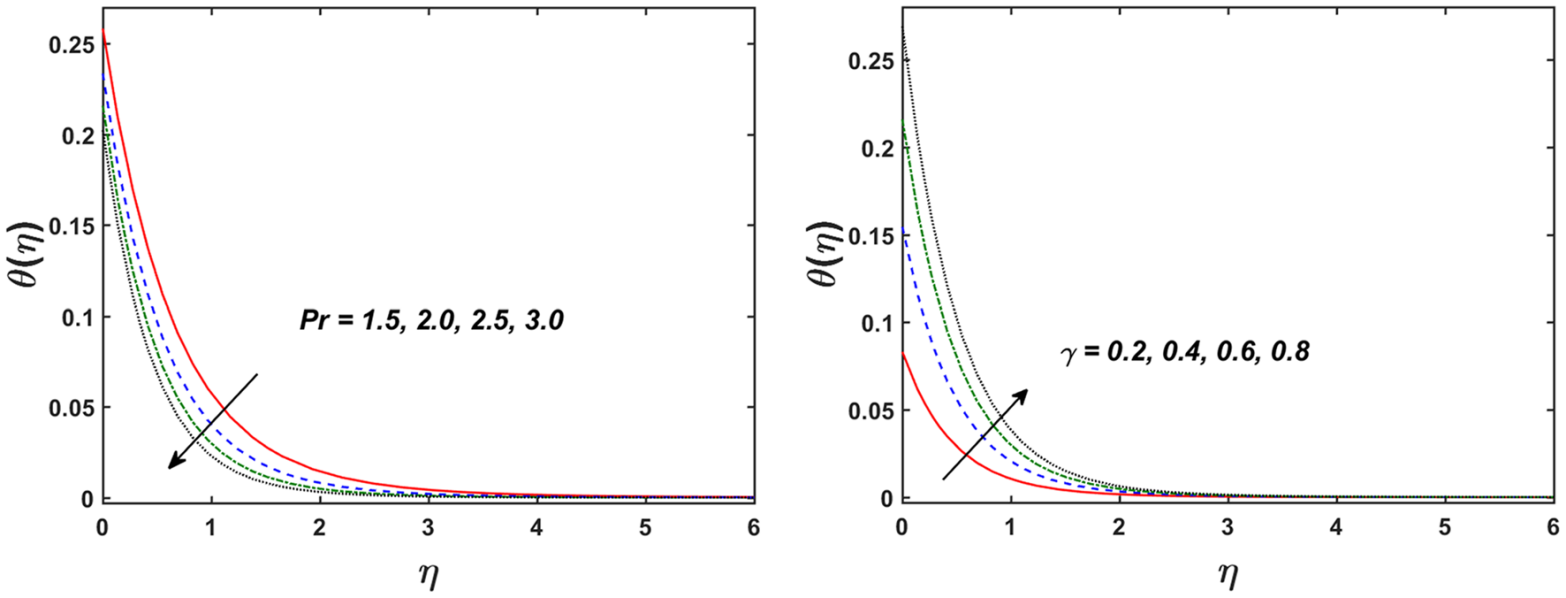
Variation in $\theta \left( \eta \right)$ for $Pr$ and $\gamma$.

**Figure 7:**
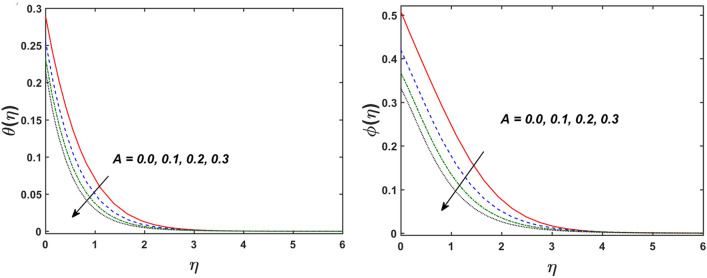
Variation in $\theta \left( \eta \right)$ and $\phi \left( \eta \right)$ for $A$.

**Figure 8:**
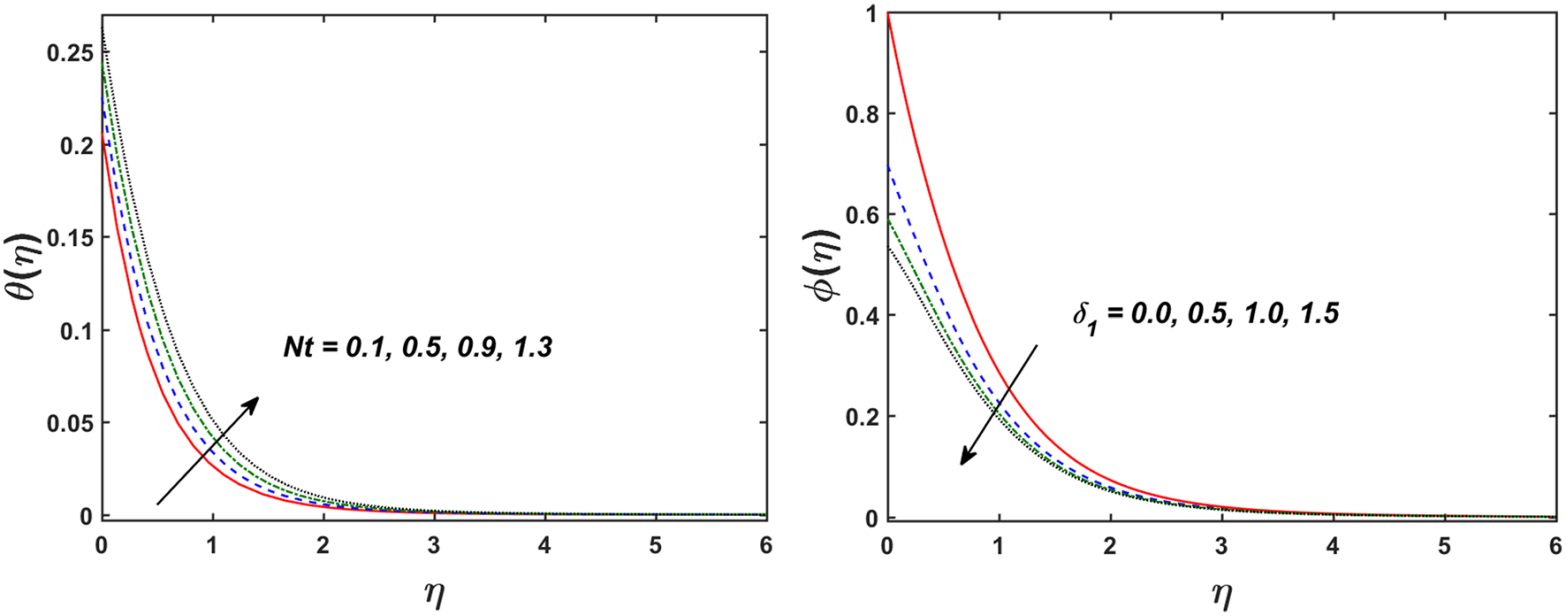
Variation in $\theta \left( \eta \right)$ for $Nt$ and $\phi \left( \eta \right)$ for $\delta_{1} .$

**Figure 9:**
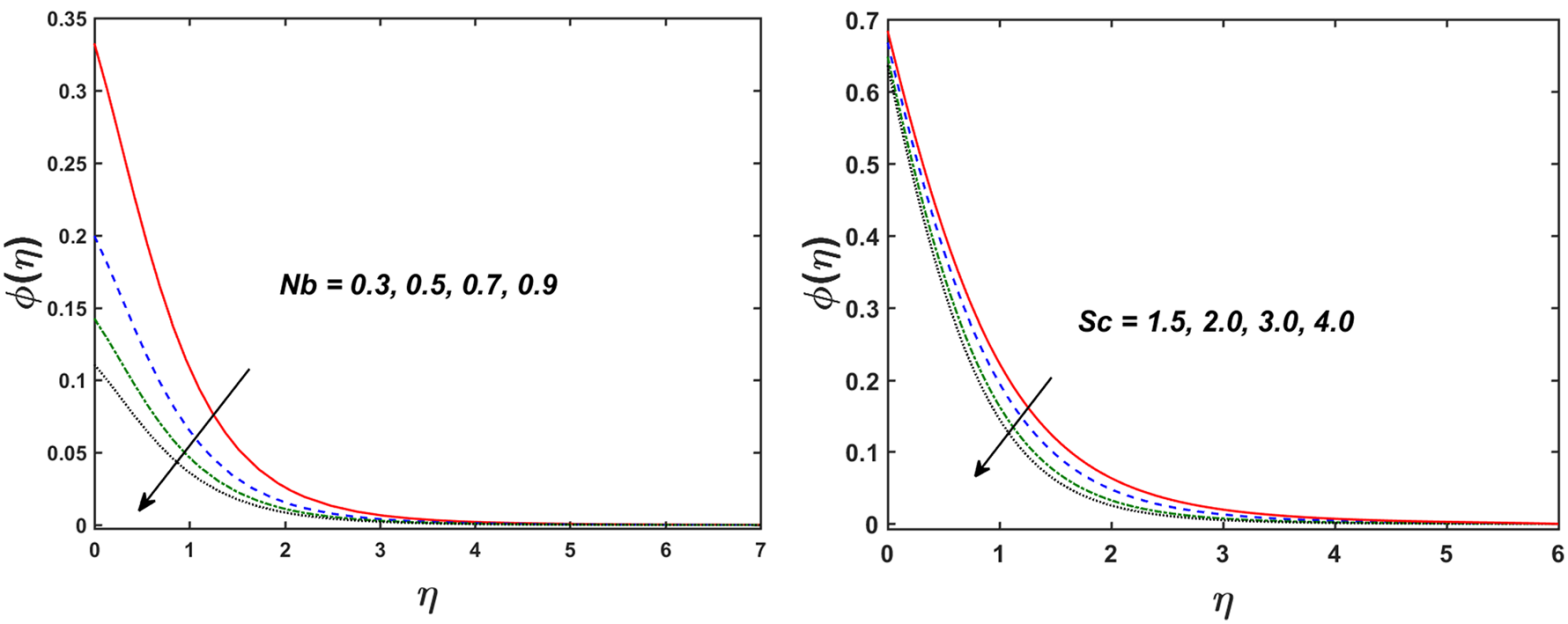
Variation in $\phi \left( \eta \right)$ for $Nb$ and $Sc.$

**Figure 10:**
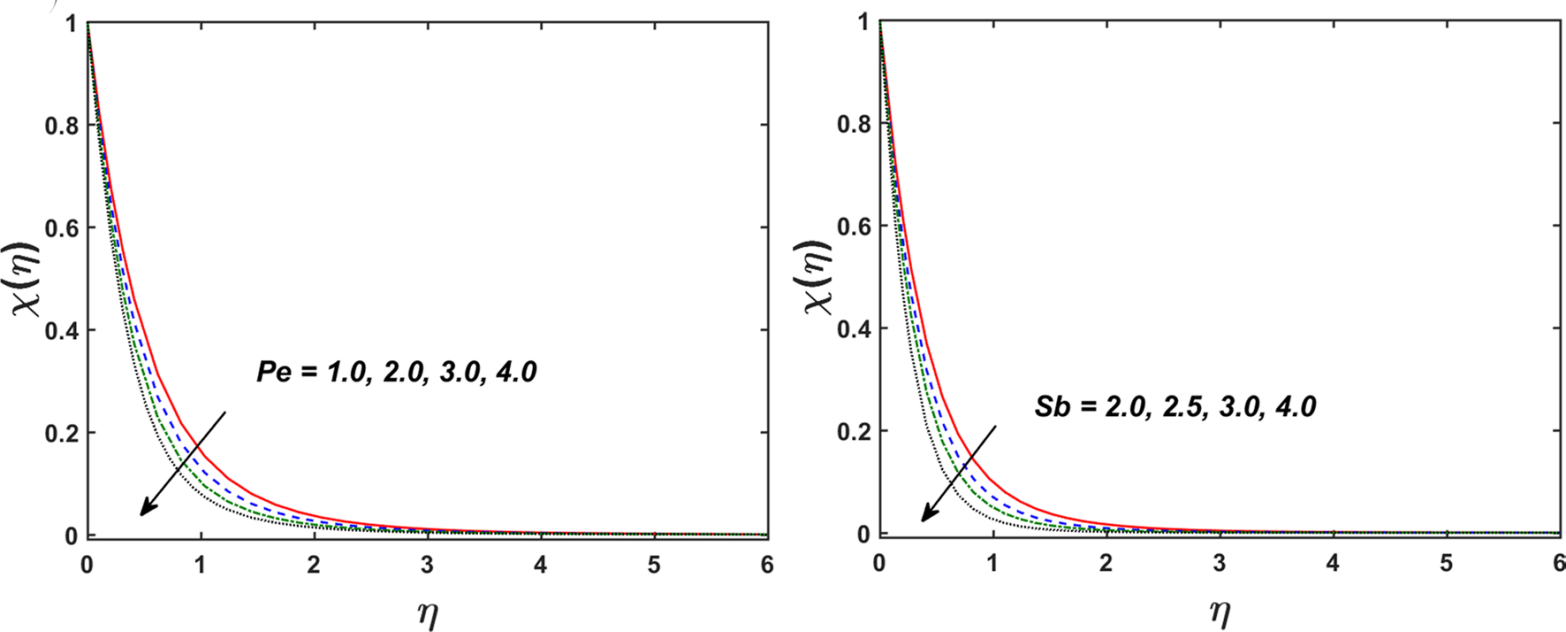
Variation in $\chi \left( \eta \right)$ for $Pe$ and $Sb$.

**Figure 11:**
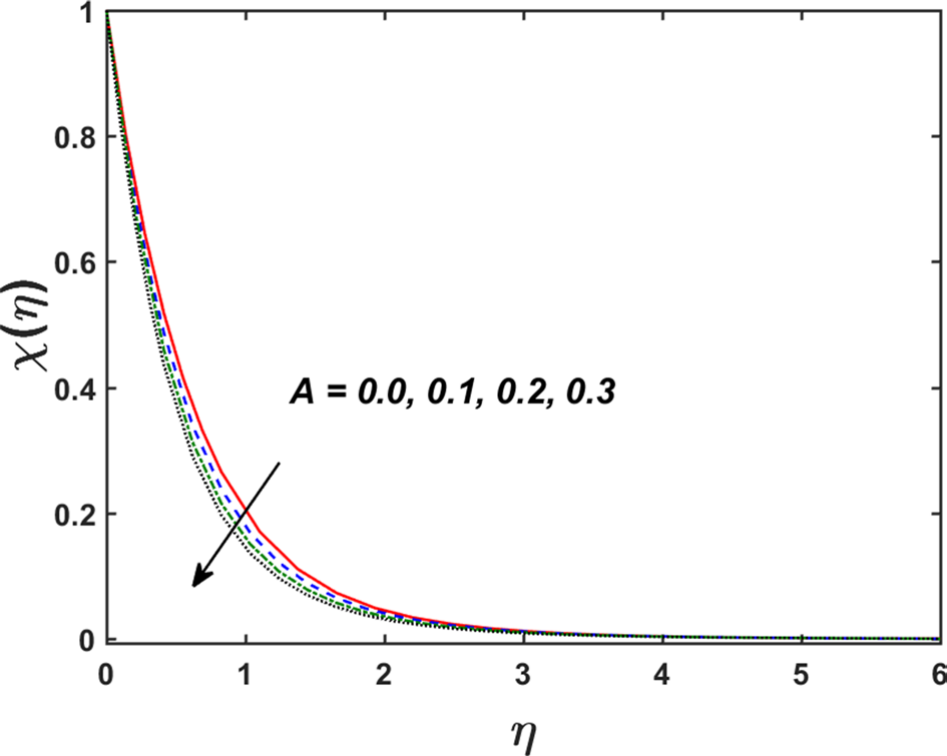
Variation in $\chi \left( \eta \right)$ for $A$ and $\pi$.

**Figure 12:**
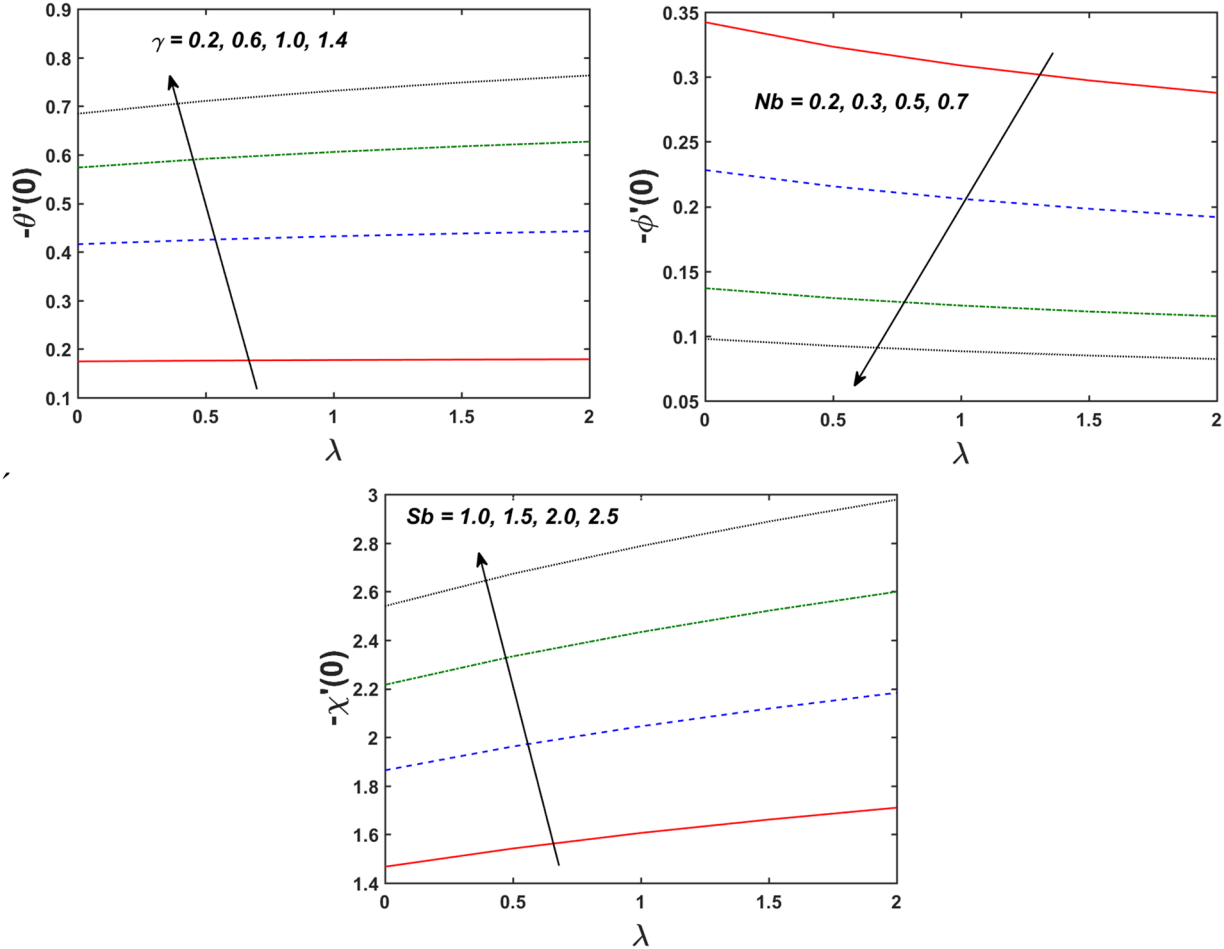
Variation of Nusselt number, Sherwood number and microorganism number for distinct parameters.

Figure 2 designates that the reduction in velocity field $f^{\prime} \left( \eta \right)$ and $g^{\prime} \left( \eta \right)$ is occurred by enlarging the values of unsteadiness parameter $\left( A \right)$. Physically, when the amount of $A$ increases, the thickness of the boundary layer decreases significantly, and this development restricts the change of laminar to turbulent flow. Hence the flow is stabilized due to the stretching sheet. The Fig. 3 exhibits the diversion in $f^{\prime} \left( \eta \right)$ and $g^{\prime} \left( \eta \right)$ sketch for various amount of time relaxation characteristic $\left( \beta \right)$. As seen in the sketched that the $f^{\prime} \left( \eta \right)$ and $g^{\prime} \left( \eta \right)$ are reduced as the $\beta$ increases. Physically $\beta$ is the ratio of relaxation time to observation time, as enlarging the $\beta$ the relaxation time also enlarges. Hence higher values of $\beta$ inhibit the fluid motion declines the $f^{\prime} \left( \eta \right)$ and $g^{\prime} \left( \eta \right)$ sketch. It is obvious that when $\beta = 0$, then viscous fluid is recovered. Further, when $\beta \ne 0$, then fluid is non-Newtonian. Figure 4 depict the influence of magnetic parameters on velocity profiles ($f^{\prime} \left( \eta \right)$, $g^{\prime} \left( \eta \right)$). Form the figures it is observed that both the velocity field diminish with larger the estimation of magnetic parameter because the Lorentz force enhances which enlarge the resistive force in a fluid. As a result, velocity of fluid decreases while the temperature profile enhances (see in Fig. 5). Figure 5 examines the temperature distribution under the impact of the Eckert number. It is seen that the temperature field and their corresponding boundary layer thickness inclines. Figure 6 point out the fluctuation in temperature profile against the various estimation of the Prandtl number. Physically, as amplifying $Pr$ the reduction is occurred in the thermal diffusivity, hence the penetration depth of temperature decays. Further, $Pr$ also control the thermal boundary layers of fluid and relative momentum thickening. It is found in Fig. 6 that the temperature distribution is expanding due to climbing the values of surface convection parameter $\left( \gamma \right)$. Physically, $\gamma$ is the proportion of the hot to colder fluid convection resistance. As $\gamma$ increases the thermal resistance fall down and hence temperature raises. The impact of unsteadiness characteristic $A$ on the concentration and temperature distribution is deliberated in the Fig. 7. The reduction occurs in temperature and concentration plots by boosting the values of $A$. The Fig. 8 examined the outcome of thermophoresis parameter $\left( {Nt} \right)$ and concentration slip parameter $(\delta_{1} )$ on the temperature sketch and concentration sketch respectively. It is seemed that the temperature sketch enlarged for greater values of $Nt$, while concentration sketch declines for the higher values of $\delta_{1}$. Physically, thermophoresis force occurs due to the temperature gradient, which leads to fast flow far off the sheet. The thermal boundary layer becomes dense by the large values of $Nt$. The diversion in concentration sketch against the distinct values of Brownian motion parameter $\left( {Nb} \right)$ is visualized in Fig. 9. It is represented that the concentration sketch shows decreasing behavior for the greater values of $Nb$. Figure 9 reported the variation in Schmidt number $\left( {Sc} \right)$ via concentration sketch. The sketch identified that the concentration sketch diminished by the enhancement of $Sc$. Physically, the ratio between viscous diffusion to molecular diffusion rate is said to be Schmidt number. The reduction is noted in Schmidt number by rising the mass diffusivity, hence the concentration sketch shrinks for $Sc$. The Fig. 10 found the impact of bio-convection Schmidt number $\left( {Sb} \right)$ and Peclet number $\left( {Pe} \right)$ via microorganism sketch. It is portrayed that the various values of $Sb$ and $Pe$, the microorganism sketch shows decreasing behavior. Physically, $Pe$ have an inverse relation with microorganism diffusivity, as microorganism diffusivity boosts then the devaluation is occurred in the microorganism density profile. The amplification in unsteadiness parameter $\left( A \right)$ shows the diminishing behavior for microorganism sketch, which is illustrated in Fig. 11. The graphical description of Nusselt number, Sherwood number, and microorganism number for several parameters is presented in Fig. 12a–c. The Fig. 12a described that as growing the values of $\lambda$ and $\gamma$ the Nusselt number shows the increasing behavior in both cases. Figure 12b clearly shows that the Sherwood number decreases for distinct values of $Nb$ and $\lambda$. Figure 12c revealed the impact of microorganism number for distinct values of $Sb$ and $\lambda$. It is portrayed that the microorganism transfer rate enhances for the higher values of $Sb$ and $\lambda$. The heat and mass transfer rate shows the effectiveness of heat and mass convection at the surface.

## Concluding remarks

The 3D MHD flow of chemically reactive Maxwell bio-convective nanomaterial liquid embedded by an exponentially extending surface in the presence of viscous dissipation and joule heating effect. The thermal and solutal energy aspect has been addressed with the influence of Brownian motion and thermophoresis effect. The thermal convective and concentration slip boundary conditions are imposed on the boundary of the sheet. The specific observation of distinct characteristics is analyzed and summarized. The main outcomes of paper are:

- By the enhancement of the magnetic characteristic and Deborah number the fluid velocity is declines due to occurrence of retardation effect.
- By the increment of the unsteadiness parameter, the fluid velocity shows diminishing behavior.
- The higher values of $Pr$ reduce the fluid temperature, while opposite trend is noted in the case of stronger $\gamma$.
- The lager estimation of the Eckert number and magnetic characteristic boost the fluid temperature.
- The effect of $A$ on temperature and concentration distribution are quantitatively similar, which is decreasing by enlarging the values of $A$.
- The concentration slip parameter shows declining behavior for higher values of $\delta_{1}$. Further slip condition finished for $\delta_{1} = 0$.
- The effects of $Sb$ and $Pe$ on microorganism distribution is qualitatively similar, which is decreasing as growing the values of $Sb$ and $Pe$.
- The tabulated results show that the Nusselt number and microorganism number increases, while Sherwood number exhibits declining behavior for higher amount of $\beta$.
- Sherwood and Nusselt number showing opposite behavior for distinct values of $Nt$.

## List of symbols

$$\left( {a,\,b} \right)$$: Stretching constants $${\text{(1/s)}}$$
$\,D_{T}$: Thermal diffusion coefficient
$$\,D_{B}$$: Mass diffusivity
$k_{0}$: Chemical reaction constant
$\theta (\eta )$: Dimensionless factor for temperature
$S$: Extra stress tensor
$f(\eta ),\,g(\eta )$: Dimensionless variables x- and y-direction
$$\left( {T_{0} ,\,C_{0} ,\,n_{0} } \right)$$: Positive constant
$T_{\infty } ,\,C_{\infty } ,\,n_{\infty }$: Ambient temperature, concentration, and microorganism
$k\left( T \right)$: Variable Thermal conductivity
$Ec_{x} ,\,Ec_{y}$: Eckert number in x- and y-direction
$$Sh_{x}$$: Sherwood number
$$Nu_{x}$$: Nusselt number
$$\,Nt$$: Thermophoresis parameter
$$\left( {u_{w} ,\,v_{w} } \right)$$: Stretching velocities $$\left( {{\text{ms}}^{ - 1} } \right)$$
$${\text{Re}}_{x}$$: Reynolds number
$T_{w} ,\,C_{w} ,\,n_{w}$: Wall temperature, concentration, and microorganism
$$\left( {u,\,v,\,w} \right)$$: Velocity components $$({\text{m}}/{\text{s}})$$
$$x,\,y,\,z$$: Space coordinates $$\left( {\text{m}} \right)$$
$$B_{0}$$: Magnetic field
$T,\,C,\,n$: Temperature, concentration, and microorganism density
$c_{p}$: Specific heat capacity
$h_{w}$: Heat transport coefficient
$$A$$: Unsteadiness parameter
$\phi (\eta )$: Dimensionless factor for concentration
$W_{c}$: Cell swimming speed
$\chi (\eta )$: Dimensionless variables for microorganism density
$\,Nb$: Brownian motion parameter
$f(\eta ),\,g(\eta )$: Dimensionless variables x- and y-direction
$$k$$: Thermal conductivity $$\left( {{\text{Wm}}^{ - 1} \,{\text{K}}^{ - 1} } \right)$$
$\,D_{m}$: Microorganism diffusion coefficient
$\left( {q_{m} ,\,j_{w} } \right)$: Heat flux and mass flux
$$Qn_{x}$$: Microorganism number
$$z_{w}$$: Microorganism flux
$$\Pr$$: Prandtl number
$$Sc$$: Schmidt number
$Sb$: Bio-convection Schmidth number
$$Pe$$: Peclet number
$H$: Concentration slip factor
$$M$$: Magnetic parameter

## Greek symbols

$$\gamma$$: Convective parameter
$\pi$: Bio-convection parameter
$$\lambda_{1}$$: Time relaxation
$$\beta$$: Deborah number
$$\rho$$: Fluid density $$({\text{kg}}/{\text{m}}^{3} )$$
$\delta_{1}$: Concentration slip parameter
$\alpha$: Thermal diffusion coefficient
$\lambda$: Stretching ratio parameter
$\tau$: Ratio of capacities
$$\sigma$$: Electrical conductivity
$${\upnu }$$: Kinematic viscosity $$({\text{m}}^{2} /{\text{s}})$$
$$\mu$$: Dynamic viscosity $$({\text{kg}}/{\text{ms}})$$

## Subscript

$w$: Surface boundary condition
$\infty$: Free stream condition
